# Supplementary material for: Evaluation of ultrasound-guided lateral thoracolumbar interfascial plane block for postoperative analgesia in lumbar spine fusion surgery: a prospective, randomized, and controlled clinical trial
Source: PeerJ. 2019 Oct 28;7:e7967. doi: 10.7717/peerj.7967 (PMC6822594; doi:10.7717/peerj.7967)
Supplement: Supplemental Information 3 [file peerj-07-7967-s003.docx]

Evaluation of ultrasound-guided lateral thoracolumbar interfascial plane block for postoperative analgesia in lumbar spine fusion surgery: A prospective, randomized, and controlled clinical trial

Background:

Thoracolumbar interfascial plane block (TLIP block), a novel regional anesthesia technique, was first performed in 2015. TLIP block effectively prevents the occurrence of pain via its action on the dorsal rami of spinal nerves. Gradually, it has become widely used in various surgical procedures. It has been reported that TLIP block (at the L3 vertebral level) provided an area of analgesia that covered the middle and had a predictable spread from L1 to S1 and from the left posterior axillary line to the right posterior axillary line in 10 participants. TLIP block can contribute significantly to a perioperative, multimodal, opioid-sparing analgesic regimen and enhance recovery time after lumbosacral spine surgery. However, there have been no reports of the application of TLIP block in randomized controlled trials of lumbosacral spine surgery.

Purpose:

We conducted a randomized controlled trial to confirm whether or not the application of TLIP block could relieve pain after lumbosacral spine fusion surgery and significantly reduce perioperative opioid consumption, as part of multimodal analgesia for patients undergoing lumbosacral spine surgery.

Methods:

The research study was performed at the First Afflicted Hospital of Anhui Medical University after approval from the local ethics committee. The trial was registered at the Chinese Clinical Trial Registry (Registration No: ChiCTR1900022233). Patients, anesthesiologists, outcome assessors and data analysts were blinded to the study intervention.

Type of study: prospective.

Estimated sample size: 50 patients.

Study period: 48 hours after surgery

Inclusion criteria: A total of 60 patients (ASA classes: I–II) who were scheduled for lumbar spinal fusion surgery.

Exclusion criteria: abnormal liver and kidney function, psychiatric disorders or use of psychiatric medications, use of anticoagulants or corticosteroids, bleeding diathesis, or a known allergy to local anesthetics.

Research procedures: Before general anesthesia was administered, the patients in Group T were injected with ropivacaine through the application of bilateral TLIP block, while the patients in Group C were injected with saline using the same technique.

The TLIP block was performed after induction of general anesthesia, as described by Hand. A high-frequency linear transducer was placed in the midline position at the third lumbar vertebra (L3), and 30 ml of 0.375% ropivacaine was injected bilaterally into the interfascial plane between the longissimus muscle (LF) and multifidus muscles (MFs) of patients in Group T. The corresponding procedure was performed using 30 ml of 0.9% saline for the patients in Group C. In our study, all TLIP block procedures were performed by the same anesthesiologists under the same medical conditions.

Standardized monitoring procedures were performed during anesthesia and surgery. Induction of general anesthesia was achieved by intravenously injecting propofol (1–2 mg/kg), sufentanil (0.3–0.5 µg/kg) and cisatracurium (0.2 mg/kg). After tracheal intubation, general anesthesia was maintained with propofol (4–10 mg/kg), remifentanil (0.25–4 µg/kg·min) and cisatracurium (0.02–0.05 mg/kg·h). By the end of skin closure, the anesthesiologists stopped the anesthetic agents and administered intravenous flurbiprofen (50 mg). After the operation, all patients were transferred to the postoperative recovery room and received PCA (sufentanil 4.5 µg/kg + 0.9% saline 150 ml, background dose 3 ml/h, self-control supplementary dose 3 ml, and locking time 10 min). BIS monitoring was performed in all patients, and BIS values were maintained at 40-60.

VAS and BCS scores were recorded at 1, 12, 24, 36, and 48 hours postoperatively. The pain score was assessed using the VAS (choices ranging from 0 [no pain] to 10 [worst imaginable pain]). The postoperative comfort scale was assessed using BCS scores (0, continuous pain; 1, painless without movement, severe pain while breathing deeply or coughing; 2, painless without movement, mild pain while breathing deeply or coughing; 3, painless when breathing deeply; 4, painless when coughing). When the VAS score exceeded 5 at rest or with movement, patients were administered intravenous sufentanil (5 µg) one or more times. The frequency of PCA compressions and remedial analgesic administration was recorded. Postoperative complications were recorded by a nurse blinded to the study groups.

Risks and benefits:

Risk: A puncture can cause local bleeding and can also lead to infection

Benefits: Patients can get better pain treatment

Observe, record and dispose of adverse events

Adverse events were collected after the subject signed the informed consent. Adverse events in the course of this test must be recorded on CRF. Adverse events need to be documented in medical terms, and the diagnosis of the disease should be given as far as possible, rather than listing the symptoms and signs. Researchers should take measures to deal with adverse events according to their needs, which should be recorded in the original medical records and CRF. All adverse events and abnormal laboratory indicators will be followed up to normal, abnormal, no clinical significance or return to pre-treatment status. The time, symptoms, degree, duration, treatment measures and outcome of any adverse events during the trial should be recorded in CRF. The correlation between the adverse events and the test drugs should be evaluated on the basis of comprehensive consideration of the complications and the combination of drugs. The researchers should record the adverse events in detail, sign and date them. If there are serious adverse events related to this study, it is necessary to fill in the report of serious adverse events, report to the head of the Department and the ethics committee at the same time, and take all measures to ensure the safety of the subjects, and provide them with relevant treatment costs and economic compensation.
